# People Lifting Patterns—A Reference Dataset for Practitioners

**DOI:** 10.3390/s21093142

**Published:** 2021-04-30

**Authors:** Konrad Kluwak, Ryszard Klempous, Zenon Chaczko, Jerzy W. Rozenblit, Marek Kulbacki

**Affiliations:** 1 Faculty of Electronics, Wroclaw University of Science and Technology, 50-370 Wroclaw, Poland; ryszard.klempous@pwr.edu.pl; 2 R&D Center, Polish-Japanese Academy of Information Technology, 02-008 Warszawa, Poland; kulbacki@pjwstk.edu.pl; 3 DIVE IN AI, 53-307 Wroclaw, Poland; Zenon.Chaczko@uts.edu.au; 4 School of Electrical and Data Engineering, University of Technology Sydney, Ultimo 2007, Australia; 5 Department of Electrical and Computer Engineering, University of Arizona, Tucson, AZ 85721, USA; jr@ece.arizona.edu; 6 Department of Surgery, University of Arizona, Tucson, AZ 85721, USA

**Keywords:** human motion dataset, ergonomics in people lifting, tag detection, human motion lab, decision support, recommending systems, data processing tag detection, motion analysis

## Abstract

Many health professionals do not use correct person transfer techniques in their daily practice. This results in damage to the paraspinal musculature over time, resulting in lower back pain and injuries. In this work, we propose an approach for the accurate multimodal measurement of people lifting and related motion patterns for ergonomic education regarding the application of correct patient transfer techniques. Several examples of person lifting were recorded and processed through accurate instrumentation and the well-defined measurements of kinematics, kinetics, surface electromyography of muscles as well as multicamera video. This resulted in a complete measurement protocol and unique reference datasets of correct and incorrect lifting schemes for caregivers and patients. This understanding of multimodal motion patterns provides insights for further independent investigations.

## 1. Introduction

The problem of maintaining correct body posture while performing lifting activities is important in many professional fields. In particular, it concerns healthcare personnel and other professionals who help people on a daily basis. The fundamental problems of proper person handling activities and setting up the most ergonomic approaches are:

- the identification of the appropriate ergonomic techniques for professional workers,
- the selection of proper ergonomic exercises for particular groups of employees,
- the determination of the quality of the performed activities, and
- the evaluation of the quality of their performance.

Therefore, to specify the correct assessment methods and ergonomic exercises, it is necessary to carry out and record both correct and incorrect person handling activities. One of the routine and often basic procedures performed by healthcare workers is patient lifting. This task requires physical exertion for prolonged periods daily. It often results in musculoskeletal disorders (MSDs) or low-back spinal disorders (LBDs) [1]. Correct lifting posture and the use of leg muscles, in particular the lateral, intermediate, and medial heads of the quadricep muscles, is a necessary condition for balancing the lumbosacral loads when manually moving a patient [2,3,4]. Although the use of equipment to lift a patient or dependent person reduces the exposure to injuries associated with manual lifting by up to 95%, it is rarely used in out-of-hospital (out-patient) settings. The purpose of this study is:

- to propose rules for building a dataset of performed activities for caregivers and dependent people,
- to use recent recording techniques, such as MoCap, EMG, and GRF to synchronously record selected activities,
- to suggest an appropriate ergonomic set of exercises for a selected group of volunteers, and
- to propose multimodal spatio-temporal motion patterns for easier identification of particular motion phases.

The most important result of this study is the observation and simultaneous registration of two mutually interacting people’s motions. We present a full correlation between the caregiver motion and patient, at the same time of the handling procedure.

In this paper, we present an example of person lifting as an action that could cause injuries to caregivers and patients. The examples of correct (safe) techniques of lifting patients demonstrate various measurable events that can occur when lifting patients in surgery. We propose a collective way of representing multi-modal movement information using Motion Tags [5,6,7,8,9,10].

The most ergonomic risk assessment methods are described in the medical literature. We also indicate works in which motion measurements and analyses were conducted, such as [11,12,13,14]. One of the tasks of manual patient transport presented in [15] is lifting the patient from a lying position to a sitting position on the edge of the bed. In the study [16] an eighty-kilogram patient dummy was used to analyze the lifting operation. The analyzed kinematic and kinetic data of the lifting process were recorded with a set of four Kinect cameras, GRF, and five EMG [17] electrodes. The lifting sequence was divided into three phases, with each triad for the ergonomic and nonergonomic versions.

In the proposed ergonomic version, attention was paid to bending the knees and straightening the back. The number of recordings made was not given. The proposal for an ergonomic approach showed less muscle activity in the lower back, at the expense of more activity for the leg muscles. The Internet of Things [18] includes important parts of wearable acquisition systems. In one paper [19] for example, the authors presented the concept and implementation of an unobtrusive wearable suit for integrated monitoring—that is, the acquisition, processing, and analysis of human motion and other physiological modalities.

In the work [15], a dummy was used for the exercises, which, as a rigid body, does not fully reflect the mechanics of the human body in the context of joint movement and muscle tension. In this work, instead of using a human dummy when simulating lifting patients, full measurements of the real person being lifted were recorded for the analysis of the patient’s kinetic and kinematic data. We focused on the important aspect of nurse–patient cooperation because, in every real situation, the patient undertakes highly individualized cooperation with the staff.

The nurse reacts to the patient’s comments related to the lifting activity performed. Experiments in which a mannequin is used do not reflect the actual situation of picking up a living person. The available mannequins are not able to reflect the real musculoskeletal system, in particular, to realistically simulate the muscle tension occurring during the movement of the patient and the nurse. The reaction and interaction to each actual patient’s lifting are individual.

**Figure 1:**
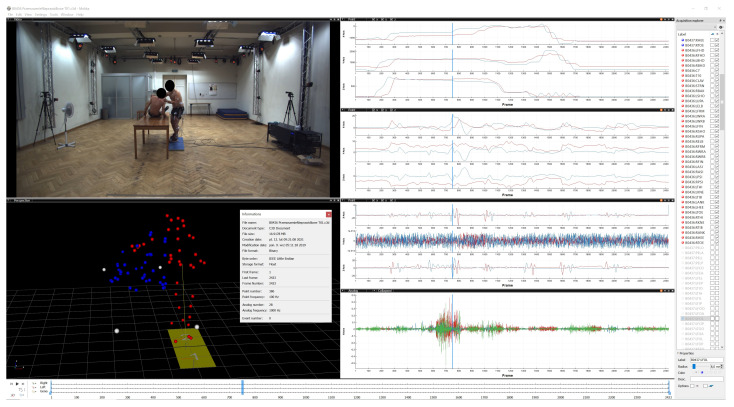
Visualization of the correct patient lifting with multimodal data (video, kinematics, kinetics, and electromyography).

This paper proposes a scheme of lifting the real patient: an experiment was carried out to show the interaction of people by recording anatomical relationships during lifting. During the patient’s lifting movement, there are interactions between the caregiver’s and the patient’s movements. Recorded multimodalities (image, kinematics, kinetics, and electromyography) provide more information at the same time than unimodal measurements do (Figure 1). Actions and interactions between the patient and the caregivers are presented. A great deal of research has been conducted into the ergonomics and safety of patient handling by medical personnel.

Multimodal measurement of the movement of two people represents complex multidimensional information with plenty of mutually dependent features. With the introduction of Motion Tags [5], we present a simple equivalent of original data emphasizing the most important features of the phases of patient lifting in multiperson interaction. Accurate instrumentation and multimodal measurements increased the efficacy at developing the assumed model. By combining measurement modalities, Motion Tags represent important information on a more abstract level and allow for efficient automation of the assessment between correct and incorrect movements.

## 2. Dataset with People Lifting Patterns (DPLP)

The Dataset with People Lifting Patterns (DPLP) was created in September 2019 during internships of students from the Wroclaw University of Technology. The recordings took place in the Human Motion Laboratory at the Research and Development Center of the Polish–Japanese Academy of Information Technology located in Bytom, Poland (http://bytom.pja.edu.pl/, accessed on 1 April 2021). The prepared dataset contains recordings of patient safe transfer from lying to sitting on a bed and squatting scenarios. These scenarios were performed by two 22-year-old male participants in two variants.

In the first case, the performances of the correct (ergonomic) transfer based on the right handling techniques [5] were registered and validated by experienced health care professionals. The second case represents an incorrect (non-ergonomic) transfer, where the motion was performed incorrectly without professional training. The squats were also performed with and without a load both in the technically correct and incorrect variants. These exercises allowed recording of the maximum muscle tension values for the tested actors. In the prepared recordings, the main attention was focused on the work of the hips, back, knees, and selected muscle tensions.

### 2.1. Measurement Configuration

Data were collected using Vicon software and 30 motion capture cameras, an EMG with a 16 channel configuration, two force plates from Noraxon, and three video Basler cameras. Multimodal registrations require hardware synchronization and hardware calibration, which are described in the paper [20]. We used the Life Science System from Vicon, which was able to acquire and synchronise all required modalities on the hardware level with synchronization based on equipment with the highest sampling rate. We stored all results on a fast VSP G200 Hitachi Data Storage System (1.5 PB capacity). Eventually, a data acquisition process required 5 GB of storage for the entirety of our experiments.

**Figure 2:**
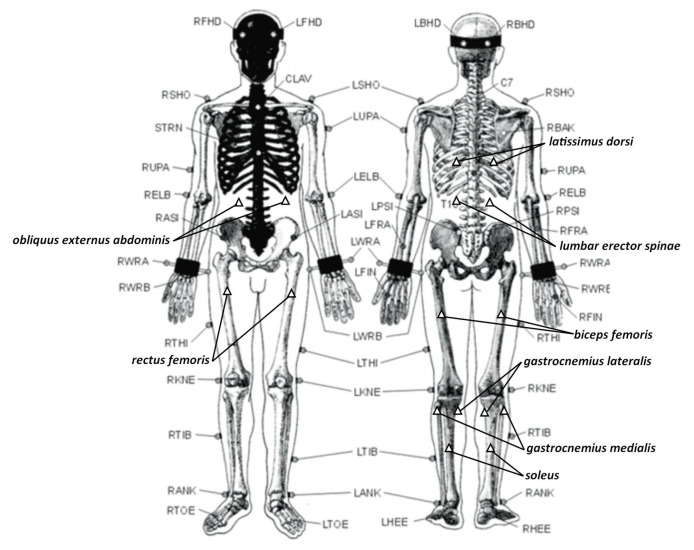
The placement of 39 motion capture markers and EMG electrodes on the nurse. The same scheme was used for the patient (without the EMGs). Skeleton schema as well as marker abbreviations of the marker names sources: http://www.lifemodeler.com/LM_Manual_2010/A_motion.shtml, accessed on 1 April 2021.

A detailed description of the applied measurement configuration was presented in [5]. The measurement devices were calibrated and synchronized (Table 1), and the system setup allowed the acquisition of 404 parameters for two people’s motions. Biometric data of the registered participants are shown in Table 2. The list and descriptions of the EMG electrode placement can be found in Table 3 and Figure 2. A description of the marker placement on the body is provided in Figure 2. Mocap markers were placed on each performer’s body (nurse and patient) as well as on the table outline (four markers) and the applied loads (two times four markers).

In addition to the data already acquired directly from the measurements, the software facilitated computing additional parameters for the body motion for each actor, such as the virtual markers, angles, moments, forces, and powers. The part of the Vicon software plug-in Gait simplified the calculation of the kinematics (angles) and kinetics (forces, moments, and powers) of the estimated joints from the applied motion model and measured positions of the XYZ markers.

In addition to the data already created from the actual measurements, the software allowed us to produce additional parameters for the body motion for each actor, such as virtual markers, angles, moments, forces, and powers. The Gait plug-in directly calculates the kinematics (angles) and kinetics (forces, moments, and powers) from the measured positions of the XYZ markers.

### 2.2. Measurement Protocols

The carryover was considered to be correct if it was performed according to the phases of movement from Table 4 and if the nurse followed the appropriate principles. Correct squats are those that abide the following rules: keep the head and neck in a proper alignment with the spine; maintain the natural curvature of the spine; do not bend at the waist (in a light squat); avoid twisting the body when moving a person; always hold a person being transferred close to your body (arms not outstretched); keep legs shoulder-width apart for balance; and use leg muscles to lift and/or pull the patient (knees should not cross the toe line). Movements of patient lifting performed by a nurse without following the given rules were considered abnormal.

### 2.3. Dataset Organization

The created dataset includes all the files that were created as a result of the session recording. It contains, in particular, **.C3D**, **.AVI**, and other files necessary for the advanced processing of recorded data. The naming convention of the files in the dataset is as described in the previous chapter. We used *Mokka*—an open-source editor for the **.C3D** data visualization. Missing marker positions were completed using Vicon Nexus software. In addition, the data **were annotated** according to the time phases present in Table 4. The annotations are visible as start and end pose labels in the **.C3D** file.

The file naming convention is as follows: **YYYY name T99.c3d**, where

- **YYYY-MM-DD** is the date of recording in the format (year, month, day);
- **name** is:
  – CorrectLifting: 3 recordings for B0436 and 3 recordings for B0437,
  – InCorrectLifting: 2 recordings for B0436 and 2 recordings for B0437,
  – InCorrectSquat: 2 recordings for B0436 and 1 recording for B0437,
  – InCorrectSquatLoad: 1 recording for B0436 and 0 recordings for B0437,
  – CorrectSquat: 1 recording for B0436 and 2 recordings for B0437,
  – CorrectSquatLoad: 1 recording for B0436 and 1 recording for B0437, and
  – InCorrectSquatLoad2: 1 recording for B0436 and 0 recordings for B0437; and
- **T99** is the individual repetition ID number of the sequence.

The dataset with patient lifting (DPLP) has been made public for scientific research purposes according to the initiative of Living Labs for Human Motion Analysis and Synthesis in Shared Economy Model [21] and is available in the resources of the R&D Center PJAIT: https://res.pja.edu.pl (accessed on 1 April 2021).

## 3. Multimodal Data Representation

In the visual analysis of the recorded data, the variable behavior of muscle activity, which depends on the movement performed, was observed. In abnormal lifting, all muscles generated high tensions simultaneously. In contrast, in the correct approach, muscle tension was distributed throughout the exercise, and activity was at a lower level. The assumptions that a correctly performed movement would decrease tension on the back muscles and increase tension on the lower extremities were confirmed.

**Figure 3:**
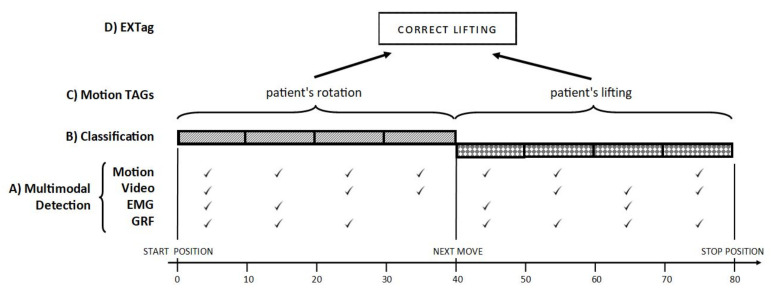
The EXTag concept with four level of operations [5].

This would result in fewer injuries to medical personnel resulting from excessive pressure on the lumbar spine. The indicated observations were also noted in the work of [5], where the concept of the Motion Tag (Figure 3) was also proposed. A Motion Tag is a concise, minimal representation of a given motion measurement segment that includes characteristic data blocks of the selected motion. In our case, the data blocks represent motion phases with a specific correlation between parameters. In the following analysis, we will attempt to determine the correctness of patient lifting motion based on Motion Tags.

Annotations of particular situations according to the measurement protocol were performed manually by the personnel involved in the recordings. The start and end positions for each movement phase were determined and marked. The proposal of the extraction of the position of the nurse and patient from markers was aimed toward an automation process that would reveal the movement phases described in Table 4.

By observing changes in the position of the patient’s pose, body markers were proposed as follows:

- F02—Approximation of the markers RWRA, RWRB, LWRA, and LWRB (the nurse’s hands) to RKNE and LKNE (the patient’s knees).
- F03—Change the values for RANK, RTOE, RHEE, LANK, LTOE, and LHEE (the patient’s feet) and RKNE and LKNE (the patient’s knees)
- F04—Change the values for RWRA and RWRB (the patient’s hand) versus RELB (the patient’s elbow).
- F05—Change the values for LWRA and LWRB (the patient’s hand) versus CLAV (the patient’s chest).
- F06—Abrupt change for most markers by 90 degrees.
- F07—Change in value of RANK, RTOE, RHEE, LANK, LTOE, and LHEE (the patient’s feet).
- F08—Change of C7, T10, and CLAV values (change of patient’s back position from horizontal to vertical).

**Figure 4:**
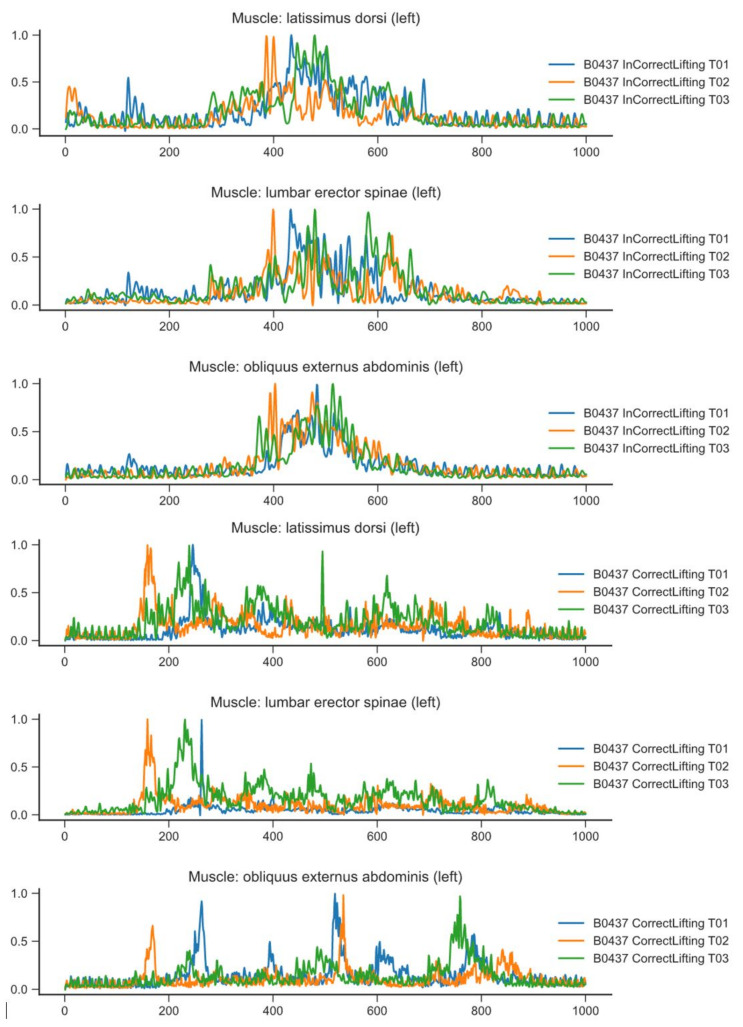
Activity of selected muscles for patient transfer. The first three graphs show the incorrect transfer, the next three graphs show the correct transfer.

**Figure 5:**
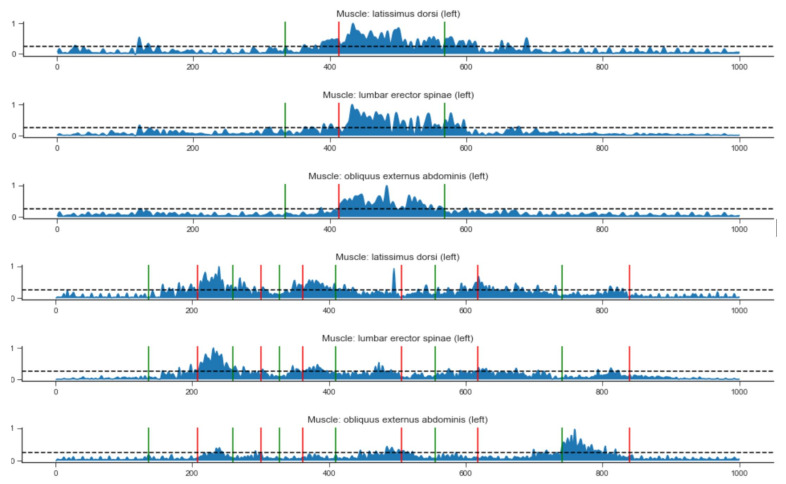
Activities of selected muscles for the patient transfer divided into movement phases (see Table 4). The first three diagrams show the incorrect transfer, and the next three show the correct transfer.

Best practices from medical protocols suggest a set of the most characteristic multimodal features for correct people handling. The individual changes in the posture of the patient in synchronization with the changes in the posture of the nurse are not sufficient to conclude that the caregiver’s movement is correct (ergonomic). Therefore, based on the additional date from the surface EMG where the muscle showed increased activity, the proper phases of the movement were chosen. The raw EMG data were rectified and normalized as in [22], and the result is presented in Figure 4. The combined multimodal motion patterns were used for the comparison of different subjects performing the movement. Each subject with selected movement phases is presented in Figure 5.

With this knowledge and the determined movement phases described in Table 4, increased muscle activity was detected and was physiologically correct according to SENIAM (The SENIAM project (Surface ElectroMyoGraphy for the Non-Invasive Assessment of Muscles) is a European concerted action in the Biomedical Health and Research Program (BIOMED II) of the European Union (http://www.seniam.org/ accessed on 1 April 2021)), recommendations were made for each specific movement phase. For each movement and processed EMG, a muscle was considered to be active only if its activity was greater than 20% of the highest tension throughout the movement. In this way, a table of muscle activity in a particular phase for each movement was obtained. If a given activity was repeated for at least three lifting samples, we considered that, in that phase, the muscle would be active for the correct movement. The described observations are included in Table 5.

## 4. Summary

The multimodal form using motion patterns presented sophisticated and detailed insights for further independent investigations of the ergonomic movements of lifting. This provided additional information regarding the mutual relations between a caregiver and patient. In addition, such a multiperson and multimodal approach reveals a new kind of relationship between different motion features (e.g., the muscle tension to the kinematic configuration of a nurse or patient in a certain phase of lifting).

We proposed and disseminated a complete measurement protocol and unique reference dataset of the correct and incorrect lifting schemes for nurses and patients. The possibility of using Motion Tags for motion correctness detection was considered as presented in [5]. Assumptions about muscle activities and changes in the marker positions were described in the paper and can be used as the basis for motion patterns.

## Figures and Tables

**Table 1:** Configuration of the custom prepared environment for multimodal data acquisition of patient lifting.

Data Type	Recording System	Recorded Parameters
Motion Capture (3D) [200 Hz]	30 Vicon cameras system (10 MX-T40, 10 Bonita, 10 Vantage)	39 markers for the nurse’s body and 39 for the patient according to the Plug-In Gait Full-Body model
Electromyography (1D) [1000 Hz]	Noraxon	16 EMG measurements (SENIAM)
GRF (3D) [1000 Hz]	Kistler Force Plates 9286BA	The direction and force of two feet
Multi-camera video (2D) [25Hz]	3 DV Basler Pilot piA1900-3gc	3 video streams (back, right, and left)

**Table 2:** Biometric data of the registered participants.

ID	Person	Role	Sex	Age	Weight [kg]	Height [mm]
0	B0436	nurse/patient	male	23–24	80	1760
1	B0437	nurse/patient	male	23–24	57	1760

**Table 3:** Placement of the EMG electrodes during the recording session (see Figure 2).

Electrode (Left Side)	Electrode (Right Side)	Muscles Name
Voltage.1	Voltage.9	latissimus dors
Voltage.2	Voltage.10	lumbar erector spinae
Voltage.3	Voltage.11	obliquus externus abdominis
Voltage.4	Voltage.12	rectus femoris
Voltage.5	Voltage.13	biceps femoris
Voltage.6	Voltage.14	gastrocnemius medialis
Voltage.7	Voltage.15	gastrocnemius lateralis
Voltage.8	Voltage.16	soleus

**Table 4:** Description of the patient lifting movement scenarios.

ID	Move	Movement Name	Movement Phases
0	E01	Correct	F01—Preparing to move
		Patients Lifting	F02— Extending hands to the patient
			F03— Patient’s leg flexion
			F04— Right arm position
			F05— Left arm position
			F06— Turning the patient over
			F07— Lowering the patient’s legs
			F08— Seating the patient
1	E02	Incorrect	F01— Preparing to move
		Patients Lifting	F02—All actions simultaneously
2	E03	Correct squats with load	F01—Squat
3	E04	Correct squats without load	F02—Upright
4	E05	Incorrect squats with load	
5	E06	Incorrect squats without load	

**Table 5:** Detected muscle (Table 3) activity in relation to movement phases (Table 4) in progress.

Muscles ID	F02	F03	F04	F05	F06	F07	F08
Voltage.1	active	active		active			
Voltage.2		active					
Voltage.3		active			active	active	active
Voltage.4							
Voltage.5			active	active	active	active	active
Voltage.6				active	active	active	active
Voltage.7	active	active		active	active	active	active
Voltage.8							
Voltage.9	active						
Voltage.10							
Voltage.11		active			active	active	active
Voltage.12	active						active
Voltage.13		active				active	
Voltage.14	active	active				active	
Voltage.15		active			active		
Voltage.16	active	active	active	active	active	active	active

## Data Availability

The dataset with patient lifting (DPLP) has been made public for scientific research purposes according to the initiative of Living Labs for Human Motion Analysis and Synthesis in Shared Economy Model and is available in the resources of the R&D Center PJAIT: https://res.pja.edu.pl, accessed on 1 April 2021.
